# Diagnostic value of direct immunofluorescence in oral mucous membrane pemphigoid: a retrospective study^؆^

**DOI:** 10.1016/j.abd.2025.501254

**Published:** 2026-01-27

**Authors:** Hongjie Jiang, Pan Wei, Zhixiu Xu, Yan Chen, Binbin Li

**Affiliations:** a Department of Oral Pathology, National Center for Stomatology, National Clinical Research Center for Oral Diseases, National Engineering Research Center of Oral Biomaterials and Digital Medical Devices, Hospital of Stomatology, Peking University, Beijing, China; b The Affiliated Stomatological Hospital of Chongqing Medical University, Chongqing Key Laboratory of Oral Diseases, Chongqing Municipal Key Laboratory of Oral Biomedical Engineering of Higher Education, Chongqing Municipal Health Commission Key Laboratory of Oral Biomedical Engineering, Chongqing, China; c Department of Oral Medicine, National Center for Stomatology, National Clinical Research Center for Oral Diseases, National Engineering Research Center of Oral Biomaterials and Digital Medical Devices, Hospital of Stomatology, Peking University, Beijing, China

**Keywords:** Autoimmune diseases, Diagnosis, Mucous membrane pemphigoid, Direct immunofluorescence, Oral mucosa

## Abstract

**Background:**

Mucous membrane pemphigoid (MMP) is an autoimmune blistering disease with heterogeneous clinical manifestations that is hard to diagnose. Direct immunofluorescence (DIF) is critical, but its role in multimodal frameworks is unclear.

**Objective:**

To assess DIF's diagnostic performance in MMP and explore factors affecting its positivity patterns in multimodal workflows.

**Methods:**

We retrospectively analyzed 79 suspected MMP patients, categorizing them into confirmed and non-confirmed groups based on clinical, histopathological, and serological criteria. DIF of perilesional mucosal biopsies showed linear deposits of IgG, IgA, IgM, C3, and fibrinogen along the basement membrane zone (BMZ). Diagnostic efficacy was assessed via ROC curve analysis.

**Results:**

55 cases were confirmed. Histopathology demonstrated subepithelial blisters in 51, with 100% specificity, 92.73% sensitivity (AUC = 0.964, p < 0.05). DIF identified 47 cases, with C3 (63.64%) and IgG (60.00%) most common, showing 85.45% sensitivity, 100% specificity, and 97.87% concordance with histopathology. Disease duration independently predicted positive IgM (p = 0.023).

**Study limitations:**

However, this study is a single-center retrospective study with a limited sample size, which has certain limitations.

**Conclusion:**

DIF, 100% specific, aids histopathology in MMP diagnosis, especially with C3/IgG linear BMZ deposition. Notably, IgM positivity correlates with prolonged disease duration, suggesting DIF efficiency may link to disease stage.

## Introduction

Mucous membrane pemphigoid (MMP), a chronic autoimmune blistering disease targeting the basement membrane zone (BMZ), poses significant diagnostic challenges due to its heterogeneous clinical presentations and overlapping features with other mucocutaneous disorders.^1^, ^2^, ^3^ While oral mucosa involvement occurs in 85% of cases, extramucosal manifestations (e.g., ocular, cutaneous) often emerge later, complicating early diagnosis.^4^, ^5^, ^6^ Current diagnostic criteria emphasize a multimodal approach integrating clinical, histopathological, and immunological evidence.^7^, ^8^, ^9^, ^10^

Histopathology remains the cornerstone of MMP diagnosis, revealing subepithelial clefting and inflammatory infiltrates at the BMZ.^6^ However, up to 30% of early-stage lesions may lack overt blistering, reducing its sensitivity.^11^ Direct Immunofluorescence (DIF) remains the gold standard for confirming MMP by detecting linear deposits of IgG, C3, IgA, or fibrinogen along the BMZ.^12^, ^13^, ^14^ Recent meta-analyses report pooled DIF sensitivity of 73%‒89% in perilesional biopsies,^15^, ^16^ with C3 and IgG exhibiting the highest positivity rates.^17^

Despite its diagnostic utility, DIF interpretation is confounded by several factors. Pre-biopsy pharmacotherapy (e.g., corticosteroids) may reduce immunoreactant deposition,^18^ while disease activity and sampling site variability (e.g., lesional vs. perilesional mucosa) impact positivity rates.^19^, ^20^ Debate continues over the optimal biopsy site, with some studies favoring non-lesional mucosa,^21^ and others supporting lesional tissue.^22^

Serological tests for anti-BP180/BP230 using ELISA show high specificity (>90%) but low sensitivity (<50%) in MMP,^23^ limiting their use as a standalone tool,^24^ Recent guidelines thus recommend combined algorithms integrating DIF, histopathology, and serology to improve diagnostic accuracy.^6^, ^25^

This study addresses critical gaps in MMP diagnostics by: 1) Systematically evaluating DIF’s performance within a multimodal framework; 2) Quantifying the impact of pre-biopsy pharmacotherapy and disease activity on DIF positivity; 3) Proposing a standardized diagnostic algorithm to optimize DIF utility in clinical practice.

## Methods

### Participants

A total of 79 patients with suspected Mucous Membrane Pemphigoid (MMP), who had undergone Direct Immunofluorescence (DIF) testing at the Department of Oral Pathology at Peking University Hospital of Stomatology between January 2023 and December 2024, were retrospectively enrolled in this study. Inclusion criteria: 1) Clinical manifestations suggestive of MMP (e.g., oral mucosal blisters/erosions); 2) Availability of complete clinical, histopathological, and DIF data. Exclusion criteria: 1) Incomplete medical records; 2) Combining with other oral mucosal diseases. For patients with multiple visits, only the first-visit data were analyzed. Control samples (n = 24) derived from patients initially suspected of MMP but excluded after comprehensive evaluation as follows. Biopsies were obtained from perilesional oral mucosa using the same protocol as confirmed cases.

### Diagnostic criteria

1 Clinical manifestations suspicious of MMP: Oral mucous membranes often present with blisters and erosions, most commonly involving the gingiva and palatal mucosa. Gingival lesions are often described as desquamative gingivitis. In addition, the lesions can involve the conjunctiva, pharynx, reproductive system, and skin, presenting with erythema, blisters, and scars.
2 Positive microscopic findings: Histopathological images show subepithelial splitting, accompanied by a non-specific mixed infiltration composed of lymphocytes, histiocytes, plasma cells, neutrophils, and eosinophils.
3 Positive serological antibody test: Positive for BP180/BP230 detected by ELISA.

According to the S3 guidelines for MMP diagnosis,^6^ antibody testing should be performed for individuals with clinical manifestations suspicious of MMP. Those with positive serological antibodies are classified into the confirmed MMP group. For individuals with negative serological examination results, if the microscopic findings are positive, they are classified into the confirmed group; otherwise, they are classified into the unconfirmed group.

### Specimen processing

Biopsy samples were submitted to the in-house pathology laboratory, embedded in OCT compound, and snap-frozen. Serial 5 μm-thick cryosections were cut using a cryostat. One section was stained with H&E for histopathological confirmation of the presence of epithelial lesions. Consecutive sections from confirmed cases were used for DIF analysis.

## DIF

Primary antibodies against IgG, IgM, IgA, fibrinogen (F), and C3 (all purchased from commercial sources, dilution according to manufacturer’s instructions) were applied to separate sections. After incubation at 37 °C for 30 minutes, unbound antibodies were removed with three PBS washes. Fluorescein Isothiocyanate (FITC)-conjugated secondary antibodies were then applied, and sections were incubated in the dark at 37 °C for another 30-minutes. Following final PBS washes, sections were mounted with antifade mounting medium and visualized under a fluorescence microscope.

DIF results were interpreted by two experienced pathologists blinded to clinical data, following established criteria:

Positive DIF: Autoantibodies (IgG, complement component C3, IgM, IgA, and fibrinogen) linearly deposit along the BMZ, visualized as continuous linear green fluorescence.

Negative DIF: Absence of linear BMZ fluorescence.

Non-specific coloring: DIF revealed discontinuous punctate/granular or focal continuous linear green fluorescence along the basement membrane zone.

### ELISA detection

Serum samples were collected at the time of biopsy. Anti-BP180 (NC16A domain) and anti-BP230 antibodies were quantified using ELISA kits (MBL International) following the manufacturer instructions. A threshold of ≥20 U/mL was defined as positive.

### Diagnostic efficiency metrics

1 Sensitivity = (Number of true positives by the diagnostic method / Number of gold standard positives) × 100%
2 Specificity = (Number of true negatives by the diagnostic method / Number of gold standard negatives) × 100%
3 Overall Agreement Rate = [(True positives + True negatives) / Total cases] × 100%
4 Positive Predictive Value (PPV) = (True positives / Total positives by the diagnostic method) × 100%
5 Negative Predictive Value (NPV) = (True negatives / Total negatives by the diagnostic method) × 100%
6 Missed Diagnosis Rate = (False negatives / Gold standard positives) × 100%
7 Misdiagnosis Rate = (False positives / Gold standard negatives) × 100%

### Statistical analysis

Statistical analysis was performed using SPSS24. Continuous variables with normal distribution and homogeneous variance were presented as mean ± Standard Deviation (SD) and compared using independent samples *t*-tests. Non-normally distributed data were reported as median and analyzed via Mann-Whitney *U* tests. Categorical data were expressed as percentages (%) and compared using Pearson’s Chi-Squared (χ²) test or Fisher’s exact test when sample sizes were small. Diagnostic performance of clinical, histopathological, and DIF findings for MMP was evaluated using Receiver Operating Characteristic (ROC) curve analysis, calculating Area Under the Curve (AUC), sensitivity, and specificity. A two-tailed p-value < 0.05 was considered statistically significant.

## Results

### Patient characteristics and serology

A total of 79 patients (22 males, 57 females; mean age 58.57 ± 11.74 years) were included. Among them, 55 patients (17 males, 38 females; mean age 61.36 ± 10.93 years) were definitively diagnosed with MMP. The oral mucosa was the predominant site of involvement (gingiva in 64.56%), with 61.82% of cases reporting a history of blistering. Disease duration ranged from 0.7 to 240 months (median 20.92 months). In MMP group, pre-biopsy medication history was documented in 61.82% of patients, with topical corticosteroids.

ELISA for BP180/BP230 showed limited utility: only 22/55 MMP patients (40.00%) tested positive for BP180, and 6/55 (10.91%) for BP230.

### Histopathology

**Fig. 1:**
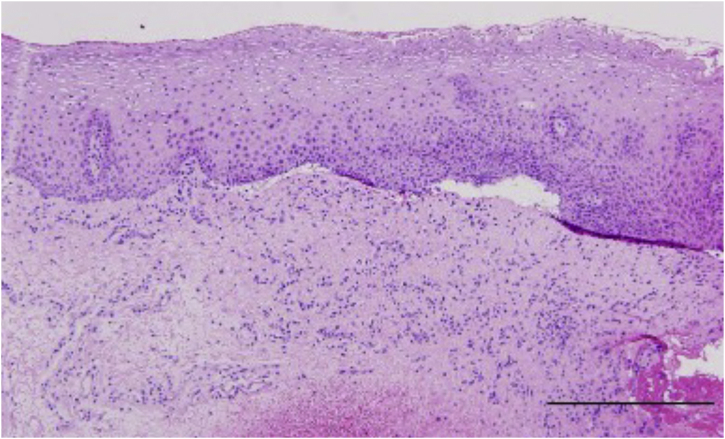
Representative histopathological features of MMP under light microscopy. Epithelial separation from the lamina propria with partial denudation. The denuded connective tissue surface appears smooth, accompanied by subepithelial blister formation. The lamina propria demonstrates infiltration of lymphocytes, plasma cells, and eosinophils.

Histopathological examination revealed subepithelial blisters in 51/55 MMP participants and in 0/24 controls, demonstrating 92.73% sensitivity, 100% specificity, and 94.94% diagnostic agreement rate (Fig. 1).

### Direct immunofluorescence findings

**Fig. 2:**
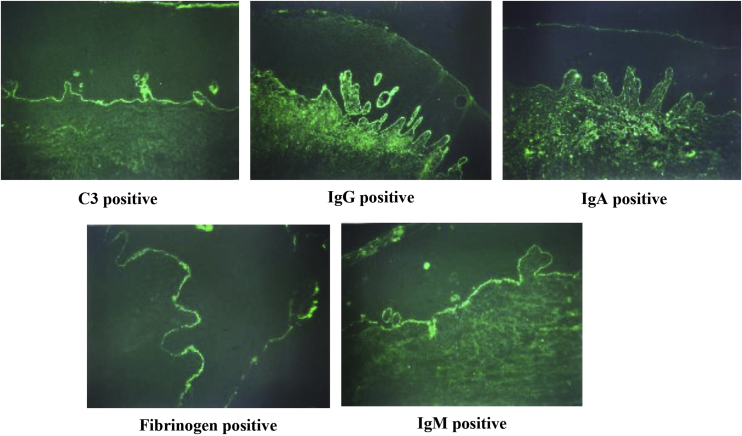
Characteristic DIF fluorescence patterns. DIF demonstrated continuous and homogeneous linear green fluorescence for C3 and IgG along the BMZ, with positive linear staining for IgA, IgM, and fibrinogen observed in a subset of cases.

In the present data, the classic DIF fluorescence pattern typically presents as a continuous, homogeneous linear green deposit along the epithelial-connective tissue junction (Fig. 2). DIF analysis revealed BMZ deposition of immunoreactants in 47/55 MMP participants and in 0/24 controls. Among DIF-positive cases, 91.49% exhibited typical homogeneous linear staining along the BMZ, while regional linear or granular fluorescence patterns were observed in 8.51% of these cases.

Notably, C3 and IgG demonstrated the highest positivity rates (65.45% and 60.00%, respectively) among DIF-positive cases. Co-deposition of C3 and IgG was observed in 45.45% of cases, whereas isolated C3 or IgG positivity occurred in 20.00% and 14.55% each. Fibrinogen, IgA, and IgM all showed positivity rates below 50%, with IgM exhibiting minimal involvement (<10%).

**Fig. 3:**
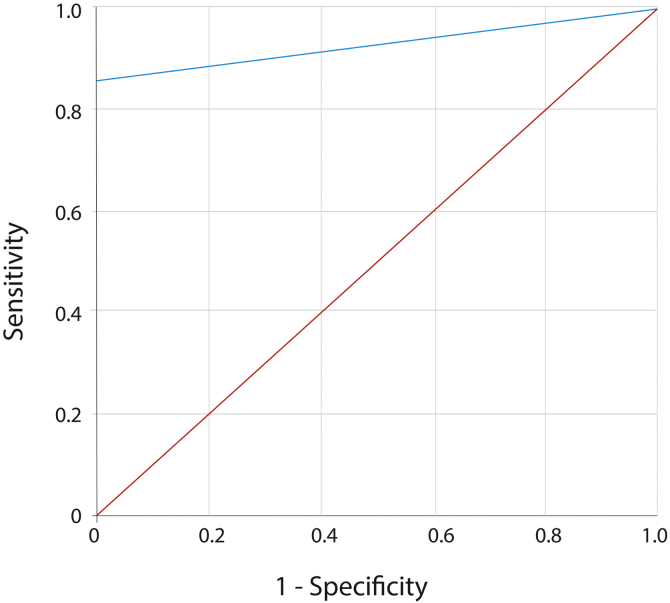
ROC curve of DIF diagnosis in MMP. ROC curve of DIF diagnosis connects coordinate points with 1 ‒ specificity (=false positive rate) as the x-axis and sensitivity as the y-axis at all cut-off values measured from the test results. The 45° diagonal line serves as the reference line, since it is the ROC curve of random classification.

The diagnostic efficacy of DIF is summarized in Table 1, with a sensitivity of 85.45%, a specificity as high as 100%, and a Youden's index of 0.8545. The AUC of DIF is 0.927 (95% CI 0.869‒0.985, p < 0.001) (Fig. 3).

**Table 1:** Diagnostic efficacy of different DIF indicators in MMP.

Examination index	Sensitivity	Specificity	Agreement rate	Positive predictive value	Negative predictive value	Missed diagnosis rate	Misdiagnosis rate
IgG	60.00%	41.67%	54.43%	70.21%	31.25%	40.00%	58.33%
C3	65.45%	66.67%	65.82%	81.82%	45.71%	34.55%	33.33%
F	45.45%	83.33%	56.96%	86.21%	40.00%	54.55%	16.67%
IgA	21.82%	95.83%	44.30%	92.31%	34.85%	78.18%	4.17%
IgM	7.27%	95.83%	34.18%	80.00%	31.08%	92.73%	4.17%
DIF	85.45%	100.00%	89.87%	100.00%	75.00%	14.55%	0.00%

In the MMP cohort, 46 of 47 DIF-positive patients (97.87%) exhibited subepithelial blisters on histopathology, demonstrating a significant correlation between these results (p *<* 0.001). The single discordant case showed scattered inflammatory cell infiltration in the lamina propria without distinct blister formation. This patient presented with recurrent oral erosions on the bilateral buccal mucosa and tongue dorsum, supported by positive autoantibody testing (both serologically and via DIF), ultimately confirming the MMP diagnosis.

Analysis of potential factors influencing DIF positivity rates revealed a significant positive correlation between disease duration and IgM positivity (p = 0.023), but showed no significant correlations with IgG (p = 0.256), IgA (p = 0.607), C3 (p = 0.541), or fibrinogen (p = 0.256). Longer disease duration correlated with IgM deposition at the BMZ (median duration: 24-months in IgM (+) vs. 3-months in IgM(-) cases; p = 0.023).

Among 55 confirmed cases, 8 patients tested negative for DIF. The clinical manifestations, histopathological features, and serological test results of these patients are presented in Table 2. The negative DIF results showed no significant correlation with the lesion sites, history of blistering, skin lesions, preoperative medication history, or disease duration (p > 0.05).

**Table 2:** Other test results of DIF-negative patients in the MMP group.

N°	Clinical manifestations	Histopathological features	Serological test results
1	Recurrent blistering and erosions on the right posterior gingiva for 1‒2 years	Subepithelial blisters	BP180(+)/BP230(+)
2	Recurrent gingiva ulcerations and erosions for over one year, with a history of blistering.	Subepithelial blisters	BP180(–)/BP230(–)
3	Recurrent ulceration and erosions on the buccal gingiva of teeth 24‒25 for the past two months, with a history of blistering.	Subepithelial blisters	BP180(+)/BP230(–)
4	Gingiva ulcers for 3‒4 years	Subepithelial blisters	BP180(–)/BP230(+)
5	Recurrent gingival ulcerations for over half a year	Subepithelial blisters	BP180(–)/BP230(–)
6	Recurrent pain and ulceration in both cheeks, lips, and tongue for over 6-years, accompanied by skin lesions on the abdomen and lower legs.	Mucosal inflammation, without evident formation of subepithelial blisters.	BP180(+)/BP230(+)
7	Recurrent buccal ulcers for three months	Mucosal ulceration with inflammation, without evident formation of subepithelial blisters.	BP180(+)/BP230(+)
8	Right buccal ulceration for over two months, with ulcers visible in the pharyngeal region.	Mucosal ulceration with inflammation, without evident formation of subepithelial blisters.	BP180(+)/BP230(–)

## Discussion

MMP is an autoimmune blistering disorder primarily affecting the mucosa and skin. The present study’s cohort demographics align with established MMP epidemiology,^10^, ^20^, ^26^, ^27^, ^28^, ^29^ affirming its predilection for elderly females and oral mucosa (gingiva > 80%).^29^, ^30^, ^31^, ^32^ Extramucosal involvement (e.g., ocular, skin) further complicates early diagnosis.^5^, ^30^

The European S3 guidelines emphasize a sequential multimodal approach for MMP diagnosis; the present study reaffirms DIF's critical role, serving as a vital complementary diagnosis tool for MMP.^6^, ^25^, ^33^, ^34^ Serologically, BP180 antibodies are detected in 46% of oral MMP cases, while BP230 reactivity occurs in 0%–40%.^35^, ^36^, ^37^, ^38^, ^39^ Histopathology may fail to detect subepithelial blisters due to technical limitations. Of particular note is the complementary role of DIF when conventional diagnostic methods yield negative results. DIF's performance corroborates meta-analyses confirming its role in multimodal diagnosis,^3^ Its near-perfect specificity and synergy with histopathology critically reduce misdiagnosis risks:^6^ it confirmed MMP in 1 histopathology-negative and 19 serology-negative cases, while excluding all non-MMP controls. This positions DIF as an indispensable adjunct to conventional methods in ambiguous presentations.

Some studies have shown that the positive rate of DIF indicators is affected by the disease course.^40^, ^41^ We propose a novel correlation: IgM deposition predicts prolonged disease duration (p = 0.023), potentially reflecting chronic antigen exposure or isotype switching in advanced MMP. This contrasts with dominant IgG/C3 responses in early stages, suggesting IgM as a candidate biomarker for disease chronicity ‒ a hypothesis warranting longitudinal serology studies.

In this cohort, only 8 confirmed MMP cases exhibited negative DIF results, aligning with literature-reported positivity rates of 80%‒95%.^3^, ^42^, ^43^ False-negative DIF outcomes primarily stem from five categories:

(i) Sampling site selection: Biopsy site selection significantly impacts DIF positivity, though optimal biopsy sites remain contentious. Current guidelines recommend perilesional tissue (0.5–1 cm around the blisters/erosions) or unaffected normal mucosa when inaccessible.^6^, ^44^ While Carey et al. report equivalent positivity rates between sites,^14^ a meta-analysis designates unaffected mucosa as optimal for MMP.^16^ Moreover, whether sampling different oral mucosal sites (e.g., gingiva, tongue, buccal mucosa) influences false-negative DIF results remains inconclusive. Certain scholars found no oral site-dependent effects,^45^ whereas others attribute this to regional variations in antigen expression and inflammatory cell distribution.^46^
(ii) Technical limitations: Suboptimal slide preparation causes false negatives, including tissue preservation failures during transport, delayed specimen processing, improper section thickness, low-quality fluorescent antibodies, or incorrect dilution ratios.^6^
(iii) Disease course: Stage-dependent immune complex dynamics lead to false-negative DIF results: insufficient deposition in early/remission phases, transient autoantibody reduction during disease fluctuations, or epidermal destruction in late-stage MMP.^41^, ^47^, ^48^
(iv) Treatment interference: Immunosuppressants (glucocorticoids or immunomodulators) suppress immune cell activity, reducing autoantibody levels, thus leading to false-negative DIF results.^40^, ^41^, ^47^, ^49^
(v) Patients/Disease heterogeneity: Suboptimal autoantibody levels in patients' serum and localized disease (e.g., isolated ocular involvement) may contribute to false-negative results in DIF testing.^50^, ^51^

Therefore, repeat biopsy is recommended for initially negative cases to improve detection.^21^, ^52^

While the protocol prioritized DIF, histopathology, and BP180/BP230 ELISA, the authors acknowledge the guideline's recommendation for IIF on salt-split skin and anti-laminin 332 testing in specific scenarios.^6^ This is particularly relevant given that 19 serology-negative MMP cases were confirmed by DIF and histopathology in the studied cohort. Incorporating IIF on salt-split skin and anti-laminin 332 testing could potentially increase serological sensitivity. Future protocols should integrate these as per S3 guidelines to optimize risk stratification.^53^, ^54^, ^55^, ^56^

This study has several inherent limitations that warrant acknowledgment. First, the single-center retrospective design inherently constrains the generalizability of findings, particularly regarding correlation analyses, treatment efficacy assessments, and long-term follow-up data. Furthermore, the limited sample size of DIF-negative cases (n = 8) precluded more robust statistical analyses, thereby restricting detailed investigation into potential confounders such as biopsy site selection or therapeutic influences.

## Conclusion

In summary, DIF is a highly specific and valuable tool for diagnosing MMP, with optimal performance when integrated with histopathological evaluation. The present findings support current guidelines advocating for DIF as a first-line diagnostic modality in clinically suspected cases. Future multicenter studies with larger cohorts are needed to explore the impact of treatment regimens and disease progression on DIF outcomes.

## ORCID IDs

Hongjie Jiang: 0009-0008-1793-0892

Pan Wei: 0000-0002-8938-6880

Zhixiu Xu: 0009-0005-1339-1340

Yan Chen: 0000-0003-2604-248X

Binbin Li: 0000-0003-0521-2945

## Authors' contributions

Hongjie Jiang: Data collection, or analysis and interpretation of data; statistical analysis; writing of the manuscript or critical review of important intellectual content; data collection, analysis and interpretation; critical review of the literature; final approval of the final version of the manuscript.

Pan Wei: Writing of the manuscript or critical review of important intellectual content; effective participation in the research guidance; intellectual participation in the propaedeutic and/or therapeutic conduct of the studied cases; critical review of the literature; final approval of the final version of the manuscript.

Zhixiu Xu: Data collection, or analysis and interpretation of data; Intellectual participation in the propaedeutic and/or therapeutic conduct of the studied cases.

Yan Chen: Statistical analysis; effective participation in the research guidance.

Binbin Li: Study concept and design; statistical analysis; writing of the manuscript or critical review of important intellectual content; data collection, analysis and interpretation; effective participation in the research guidance; critical review of the literature; final approval of the final version of the manuscript.

## Financial support

None declared.

## Research data availability

The entire dataset supporting the results of this study was published in this article.

## Conflicts of interest

None declared.
